# Focusing light through multimode fibres using a digital micromirror device: a comparison study of non-holographic approaches

**DOI:** 10.1364/OE.420718

**Published:** 2021-05-10

**Authors:** Tianrui Zhao, Sebastien Ourselin, Tom Vercauteren, Wenfeng Xia

**Affiliations:** School of Biomedical Engineering and Imaging Sciences, King’s College London, 4^th^ Floor, Lambeth Wing St Thomas’ Hospital, London SE1 7EH, United Kingdom

## Abstract

Focusing light through a multimode fibre (MMF) has attracted significant research interest, mainly driven by the need for miniature endoscopes in biomedicine. In recent years, digital micromirror devices (DMD) have become increasingly popular as a high-speed alternative to liquid-crystal spatial light modulators for light focusing via wavefront shaping based on binary amplitude modulations. To exploit the potentials and limitations of the state-of-the-art DMD-based wavefront shaping methods, in this study, for the first time, we compared four representative, non-holographic and DMD-based methods that are reported so far in literature with the same experimental and simulation conditions, including a real-valued intensity transmission matrix (RVITM)-based algorithm, a complex-valued transmission matrix (TM)-based algorithm, a conditional probability algorithm and a genetic algorithm. We investigated the maximum achievable peak-to-background ratio (PBR) in comparison to theoretical expectations, and further improved the performance of the RVITM-based method. With both numerical simulations and experiments, we found that the genetic algorithm offered the highest PBR but suffered from the lowest focusing speed, while the RVITM-based algorithm provided a comparable PBR to that of the genetic algorithm, and the highest focusing speed.

## 1 Introduction

Focusing light through MMFs has been an area of increasing interest as it enables high-resolution images of internal organs and tissues to be acquired at a fibre tip with rich optical contrast. Compared to multi-core coherent fibre bundles that are commonly used in biomedical endoscopy, MMF-based endoscopy benefits from a greater degree of miniaturisation, a higher spatial resolution and a lower cost [1–3]. In recent years, wavefront shaping (WS) emerged as an effective way of controlling light transport through MMFs [4–8]. In WS, the wavefront of the incident light is modulated to correct for the mode dispersion-induced wavefront distortions so that the waves interfere constructively at the desired location through a scattering medium (such as a MMF) to form a tight optical focus. As such, an optical focus can be raster-scanned over the tip of a MMF to interrogate tissue in a wide range of optical microscopy modalities, including confocal [9], fluorescence [2, 10–12], two-photon [13], Raman [14, 15] and photoacoustic microscopy [16–18].

A number of methods have been proposed for WS in the past decade [1, 3, 7, 9, 11, 19–25]. Liquid-crystal spatial light modulators (LC-SLM) were popularly employed in WS to provide phase modulations [1, 4, 6, 7]. Early WS works focused on iterative algorithms that used light intensity at the target position as feedback; the incident wavefront was then iteratively optimised by maximising the strength of feedback signal [4, 26]. Digital optical phase conjugation (DOPC) was also studied for WS with a LC-SLM [7], in which a laser beam was focused at the target position at one end of a MMF and the conjugated field of the transmitted light at the other end was projected back to focus at the original focusing point. TM-based methods were also studied [1, 3, 6, 27, 28], in which the light transport characteristics of a disordered medium was modelled as a complex-valued TM, which was then estimated in a characterisation measurement. Subsequently an optimal input field was determined to correct for the light field changes according to the estimated TM to generate an optical focus. In a study by Zahra Fayyaz et al. [29], the performance of various algorithms with phase modulations was compared in numerical simulations including a continuous sequential algorithm, a partitioning algorithm, a TM estimation method, a particle swarm optimization algorithm, a genetic algorithm (GA), and a simulated annealing algorithm. However, although demonstrated effective in producing high optical enhancement at the focusing position through disorder media, the speed of these methods is usually limited by the slow frame rates of LC-SLMs (typically ~100 Hz).

As a high-speed alternative, DMDs have been studied intensively for WS [2, 11, 20–24] in the last few years as they possess a much higher frame rate (23 kHz). A DMD comprises a large array of micromirrors; each micromirror can be independently switched between two statuses (‘ON’ and ‘OFF’) and hence provides binary amplitude modulations. Iterative algorithms [30, 31], DOPC [32] and TM methods [2, 11, 20, 33] have also been demonstrated with binary amplitude modulations for focusing light through optical diffusers. Similar to LC-SLM, with the iterative algorithms, an optimal DMD pattern was determined via iterative optimisation of feedback signals. However, DOPC and TM methods with binary modulations using a DMD have additional complexities compared to those with a LC-SLM; the conjugated field of the transmitted light and the optimal input field were first converted into binary patterns using intensity thresholding and then displayed using a DMD for focusing, respectively. In MMF characterisation, holographic methods are often used to retrieve phase information from the output optical speckles [1, 3, 6] using an optical reference arm that increases the system complexity and degrades its temporal stability. To address this issue, non-holographic methods have been investigated in recent years. Iterative optimisation algorithms were proposed to calculate a complex-valued TM from intensity-only input-output pairs [20, 27, 28]. Algorithms to directly calculate the real part of the complex-valued TM were also reported to form a binary transmission matrix to directly determine an optimal DMD patterns for focusing at output positions [22, 23]. Another method calculates the conditional probability of switching ‘ON’ each micromirror for causing light focusing at the target position, and produces an optimal DMD pattern by setting a threshold to switch ‘ON’ micromirrors with higher probabilities [21]. In our previous works [24, 25], we developed a high-speed method that characterises the light intensity changes through a disordered medium as a RVITM, based on which an optimal DMD pattern can be determined for light focusing through an optical diffuser [25].

Although these methods have shown effectiveness for focusing light through scattering media, direct performance comparison of non-holographic DMD-based methods on a single experimental or simulation platform has not been extensively studied so far in literature. In this work, we studied different categories of prominent WS methods that have been used to focus light through MMFs using a DMD, with the aim to exploit the potentials and limitations of the existing DMD-based WS methods for endoscopy applications. Here we focus on non-holographic methods as they benefit from a simpler setup and a higher temporal stability compared to holographic methods, including the RVITM algorithm [25], a phase retrieval algorithm to estimate a complex-valued TM [20], the conditional probability algorithm (CPA) [21] and a genetic algorithm (GA) [19, 31] as a representative of iterative algorithms. We investigated the theoretical PBRs with different criteria for the generation of the optimal DMD patterns in simulation and further increased the PBRs in both simulation and experimental tests. Importantly, we demonstrated light focusing through a multimode fibre using the RVITM method for endoscopy applications, and improved the maximum PBR of the RVITM algorithm by setting a new threshold for determining the optimal DMD pattern for light focusing.

## 2 Methods and materials

### 2.1 Theoretical peak-to-background ratio

**Fig. 1:**
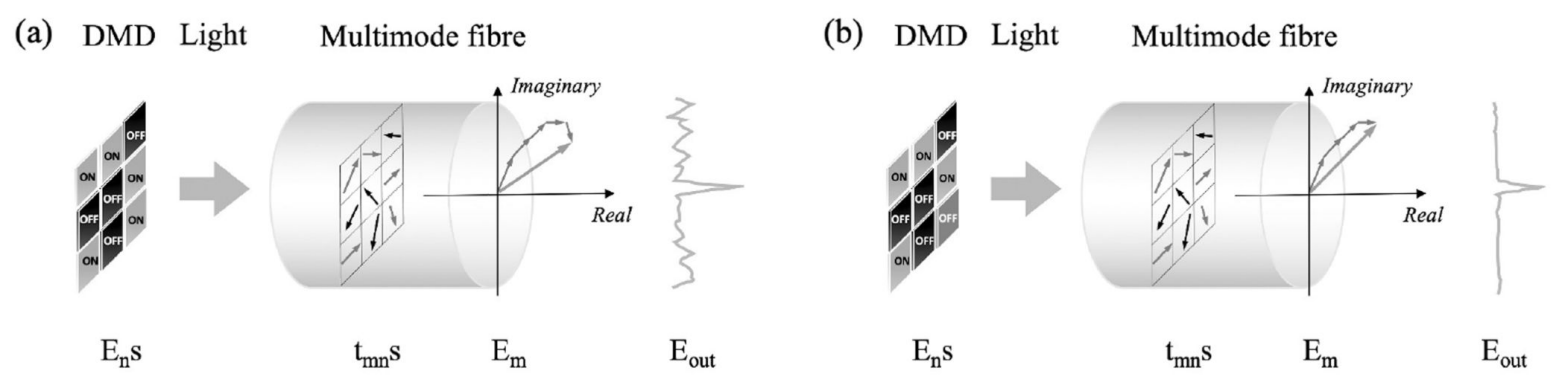
Schematic diagrams of the principle of wavefront shaping using binary amplitude modulations with a digital micromirror device (DMD). (a) When micromirrors producing light field components with phases in the range of [−*π*/2 + *θ_R_*, *π*/2 + *θ_R_*] (represented by red arrows) are switched ‘ON’, constructive interference of light fields at the target output position forms a light focus. (b) Switching ‘OFF’ those micromirrors (marked as blue) that contributed more to the background than the focusing position further improves the peak-to-background ratio.

In binary amplitude modulations with a DMD, the amplitude of the incident light field (A) at the DMD plane is considered to be uniform. Thus, switching ‘ON’ the *n^th^* micromirror leads to a contribution to the *m^th^* output mode by *At_mn_*, where *t_mn_* is a transmission constant in the TM representing the phase and amplitude changes. To produce a tightly focused light spot, constructive interference at the target output position is required and hence those micromirrors with *t_mn_* representing a phase change within [-*π*/2 + *θ_R_*, *π*/2 + *θ_R_*] are required to be switched ‘ON’, where *θ_r_* is the phase of a reference light field. For simplicity, *θ_R_* is usually chosen as 0, leading to *Re*(*t_mn_*) > 0 as the commonly used criteria for determining the switched ‘ON’ micromirrors to maximise the light intensity at the target output position for focusing (Fig. 1a) [22, 32]. However, each switched ‘ON’ micromirror also contributes to the background light intensity and some of the micromirrors may contribute more energy to the light intensity of the background than that of the target position. Thus, the highest PBR is not guaranteed by using *Re*(*t_mn_*) > 0 as the criteria. As such, switching ‘OFF’ those micromirrors that contributes substantially to the background light intensity can lead to a higher PBR (Fig. 1b).

In a study by Wang *et al*. [32], the ensemble average of the peak output intensity with binary modulations using a DMD can be expressed as:

(1) $$I_{p}=2NA^{2}\sigma^{2}\left(\frac{\sin2\varphi /2+\varphi }{2\pi }\right)+\frac{\pi }{2}N\left(N-1\right)A^{2}\sigma^{2}{\left(\frac{\sin\varphi }{\pi }\right)}^{2}$$

in which *t_mn_* is assumed to obey a Rayleigh distribution and |*t_mn_*|^2^ follows an exponential distribution |*t_mn_*|^2^ ~ *e* ^−1/2*σ*^ with 2*σ* ^2^ as the ensemble average intensity of each element, *φ* is the upper bound of absolute phase difference: 0 ≤ |Δ*θ*| < *φ*. The average of the background intensity is expressed as:

(2) $$I_{b}=2NA^{2}\sigma^{2}\frac{\varphi }{\pi }$$

So, the theoretical PBR is estimated as:

(3) $$PBR=\frac{I_{p}}{I_{b}}=\frac{2NA^{2}\sigma^{2}\left(\frac{\sin2\varphi /2+\varphi }{2\pi }\right)+\frac{\pi }{2}N\left(N-1\right)A^{2}\sigma^{2}{\left(\frac{\sin\varphi }{\pi }\right)}^{2}}{2NA^{2}\sigma^{2}\frac{\varphi }{\pi }}\approx \frac{N\;\sin^{2}\varphi }{4\varphi }$$

Thus, the theoretical PBR is a function of the upper bound of the absolute phase difference *φ*. When *φ* = *π*/2, the intensity at the *m^th^* output mode reaches the maximum, while the PBR is the highest at *φ* = 0.371*π*. However, this formula only considers phase information for producing the optimal input pattern for light focusing, while there are algorithms that are not solely based on phase information for determining the optimal DMD patterns, such as the RVITM algorithm, GA and CPA. As the PBR is considered as a more accurate performance metric than the peak intensity at the focus for quantifying the quality of light focusing and various imaging applications, in the following sections, we studied the maximum achievable PBR for different non-holographic methods in comparison to the theoretical values that can be achieved using only phase information for the optimal DMD pattern determination.

### 2.2 DMD-based non-holographic algorithms

#### RVITM-based algorithm

The relationship between the input and output light intensity distributions of a disordered medium such as a MMF can be approximated by a RVITM, which indicates the contribution of each micromirror to the light intensity at the target output position. In previous studies, we demonstrated that binary and grayscale images that were projected into a MMF could be faithfully retrieved using a RVITM [24], and high-speed photoacoustic-guided WS through an optical diffuser with a total runtime of 300 ms [25]. Several WS methods based on similar principles were also reported [22, 23, 34]. Briefly, a Hadamard matrix H ∈ (-1, +1) with dimensions of N × N was used to construct two binary matrices *H*_1_ = (*H* + 1)/2 and *H*_2_ = (−*H* + 1)/2. These 2*N* binary Hadamard patterns ([*H*_1_, *H*_2_]) were sequentially displayed on the DMD whilst the output speckles at the distal fibre tip were recorded by a camera. The relationship between the input and output light intensities can be modelled as:

(4) $$\left[\begin{array}{ccc} I_{1}^{1} & \cdots & I_{1}^{2N}\\ \vdots & \ddots & \vdots \\ I_{m}^{1} & \cdots & I_{m}^{2N} \end{array}\right]=RVITM•\left[H_{1},H_{2}\right],$$

and thus the RVITM can be calculated via:

(5) $$RVITM\;=\;\left[\begin{array}{ccc} 2I_{1}^{1}-I_{1}^{1} & \cdots & 2I_{1}^{2N}-I_{1}^{1}\\ \vdots & \ddots & \vdots \\ 2I_{m}^{1}-I_{m}^{1} & \cdots & 2I_{m}^{2N}-I_{m}^{1} \end{array}\right]•{\left[H,-H\right]}^{T},$$

where $I_{m}^{k}$ is the intensity at the *m^th^* output position when the *k^th^* binary Hadamard pattern is displayed as input, *k* ∈ (0, · · ·, 2*N*). Accordingly, the transmission constant element connecting the *m^th^* output mode and the *n^th^* input mode was calculated as: $rvit_{mn}=\left(1/N\right)\Sigma_{k=2N}I_{m}^{k}h_{n}^{k}$, where $h_{n}^{k}$ is the *n^th^* element in the *k^th^* Hadamard pattern. *rvit_mn_* can be further expressed to encode both the phase and amplitude information of the corresponding complex-valued *t_mn_* as [25]:

(6) $$rvit_{mn}=A_{mn}A_{R}\cos\left(\theta_{mn}-\phi_{R}\right)$$

where *Amn* = |*t_mn_*| is the amplitude of *t_mn_*, *θ_mn_* = *arg*(*t_mn_*) is the phase of *t_mn_*, *A_R_* and *ϕ_R_* are the amplitude and phase of the output light field when all micromirrors are switched ‘ON’, respectively. So, a positive rvi*t_mn_* indicates that a phase difference between *θ_mn_* and *ϕ_R_* is in the range of [−*π*/2,*π*/2] and therefore, switching ‘ON’ all the micromirrors with positive *rvit_mn_* values maximises the light intensity at the *m^th^* output mode as demonstrated in previous studies [22, 23, 25, 34]. Furthermore, as *rvit_mn_* represents intensity contributions [24], we hypothesize that some of the micromirrors with small *rvit_mn_* values may contribute more to the intensity of the background than that of the focus. To test our hypothesis, all the micromirrors were ranked in descending order based on their corresponding *rvit_mn_* values, and different groups of micromirrors with top P (varying from 1 to 100%) of the *rvit_mn_* values were switched ‘ON’ for focusing in both experiments and numerical simulations. A PBR was then calculated for each P value to study the dependency of the PBR on the P value.

#### Estimated TM-based algorithm

A reference-less method well described in Ref. [20] was used for TM estimation. A set of random binary patterns with 50% micromirrors ‘ON’ were displayed on a DMD whilst the speckle intensities behind a MMF were captured by a camera. The total number of patterns was set to be 6N to ensure a high-quality TM estimation, where N is the total number of independent micromirrors used for light modulation. A Bayesian phase retrieval algorithm [35] was then used to calculate a complex-valued TM from intensity-only input and output pairs via iterative optimisation. This algorithm was chosen for the TM estimation as it had been demonstrated with a DMD-based setup and it benefits from a moderate computational cost [20]. An open-source script of the phase retrieval algorithm [35] was used in this work. A total number of 200 iterations were used because it was found that the results were converged after about 200 iterations. As this algorithm provides phase values of the TM, optimal DMD patterns with both |*arg*(*t_mn_*)| < *π*/2 (*Re*(*t_mn_* > 0)) and |*arg*(*t_mn_*)| < 0.371*π* were used for focusing and their focusing performance were compared.

#### Conditional probability-based algorithm

The CPA was described in detail in Ref. [21]. Briefly, a total number of 6N random binary patterns were used as inputs, whilst the intensities of speckles at the output of a MMF were captured. There were three steps involved in the generation of an input DMD pattern for focusing. First, an intensity threshold was used to divide the output intensities into two groups: a ‘focusing’ and a ‘non-focusing’ group. Second, the conditional probability of the fact that switching ‘ON’ each micromirror leads to light focusing at the target output position (‘focusing’ group) was calculated via the Bayes’ theorem. Finally, a threshold was used to produce the optimal DMD pattern for light focusing through the MMF by switching ‘ON’ micromirrors with a conditional probability higher than the threshold. In this work, in order to obtain the maximum PBR, the first threshold was set as the 80 percentile of all intensities at the target position and the second threshold was set as the median value of all probability values, which was demonstrated in Ref. [21] with the highest experimentally achieved enhancement.

#### Genetic algorithm

The method for implementing a GA for light focusing through a diffuser was detailed in Ref. [19, 31]. In this work, we used the same process but employed the PBR of the output light field as the feedback to be maximised other than the intensity at the target position. First, a total number of 20 random binary patterns with approximate 50% micromirrors ‘ON’ were used as the 1^*st*^ generation population, each pattern is considered as the chromosome of an individual, the state of a micromirror was considered to be a chromosome code (‘1’ for ‘ON’ and ‘0’ for ‘OFF’). Output speckles intensities were recorded when displaying these binary patterns on a DMD and their PBR values were compared. Individuals in the 1^*st*^ generation population were ranked according to their corresponding PBRs in the outputs. Then individuals with larger PBRs were assigned larger probabilities to be selected as parents to produce the next generation population by crossing the parent chromosomes with a constant cross rate. Mutation was also introduced by randomly switching a small number of chromosome codes with a mutate rate to avoid locally optimal solutions. In the next step, the new generation was ranked according to the resulting PBRs and produced the next generation patterns through the aforementioned progress. After a large number of iterations, the chromosome codes leading to a high PBR were saved in the optimal DMD pattern in the new generation. In numerical simulations, 30000 generations were implemented and the cross rate was set to be 0.6 and the mutate rate, 0.02. In experiments, 4000 generations were implemented, and the mutate rate was set to be 0.1 * *e* ^−*G*/600^ + 0.02 to speed up the optimisation, where G is the index of the generation.

### 2.3 Numerical simulation

Numerical simulations were implemented in MATLAB to investigate the performance of different algorithms described in Sec. 2.2. A complex-valued TM was generated with random phases and amplitudes following an uniform and a Rayleigh probability density function [4] between 0 and 2*π*, and 0 and 1, respectively. The number of input micromirrors (N) was set to be 32 × 32 while the number of output pixels (M) was set to be 64 × 64. Output light intensities were calculated based on the simulated TM via $E_{m}={\left|\Sigma_{n=1}^{N}\;t_{mn}E_{n}\right|}^{2}$, which were then fed to those algorithms for comparison. The resulting PBR was calculated as the ratio of the intensity at the focusing pixel over the average intensity in the background, for the evaluation and comparison of the focusing performance with different algorithms. In addition, since the TM for simulating the MMF was known, it was used as a ground truth for comparison with results achieved with DMD-based algorithms.

As the TM elements follow a circular Gaussian distribution [4], modulating phases of output light field components coming from all input modes to an ideal phase *ϕ* = 0 or to *ϕ* = *ϕ_R_* leads to approximately the same constructive interference at the output position. For the ease of comparison with the RVITM algorithm which employs *ϕ_R_* as the ideal phase, the focusing condition was chosen as |*θ_mn_* − *ϕ_R_*| < *φ* rather than |*θ_mn_*| < *φ*. Both *φ* = *π*/2 and *φ* = 0.371*π* were used as the upper boundary for producing the DMD pattern to focus light in the TM-based approaches.

### 2.4 Experimental setup

**Fig. 2:**
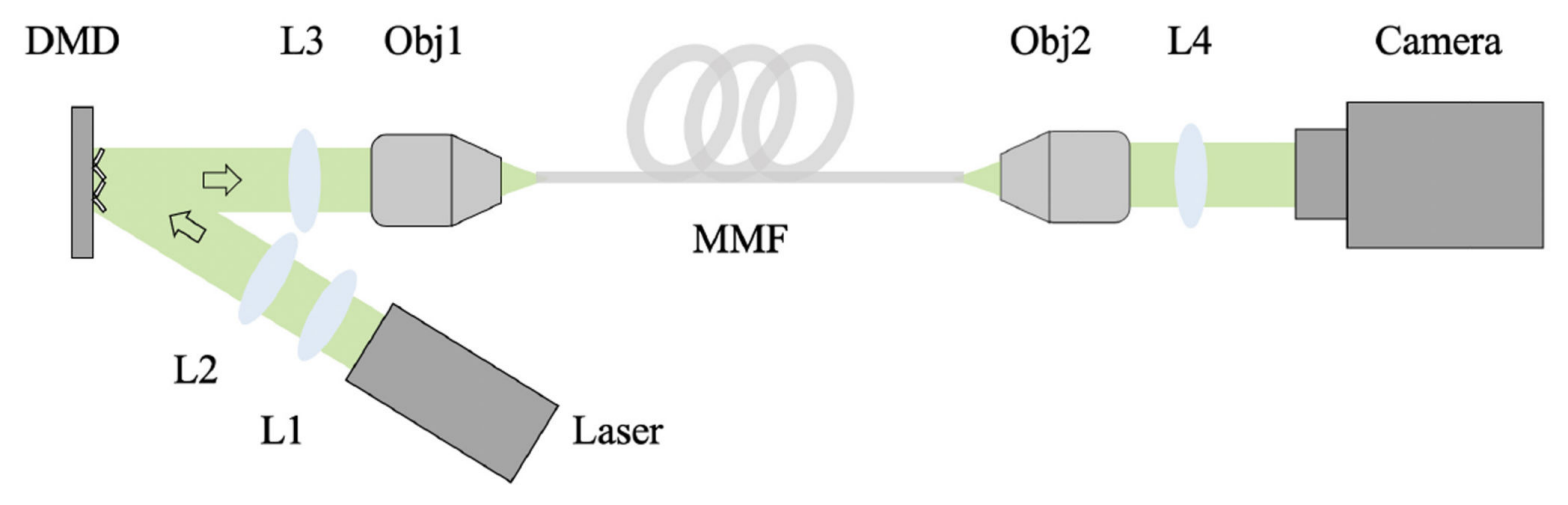
Schematic of the experimental setup. L1-L4, convex lenses; Obj1-Obj2, 20× objectives; DMD, digital micromirror device; MMF, multimode fiber.

The experimental setup is illustrated in Fig. 2. The light source was a collimated diode-pumped solid-state laser module (532 nm, 4.5 mW, CPS532, Thorlabs, New Jersey, USA). After beam expansion through two achromatic lenses (AC254-030-A-ML; AC254-075-A-ML, Thorlabs, New Jersey, USA), the laser light was spatially modulated using a DMD (DLP7000, Texas Instruments, Texas, USA) and then projected onto the proximal facet of a MMF (105 μm, 0.22 NA, 1 m, M43L01, Thorlabs, New Jersey, USA) via a tube lens (AC254-050-A-ML, Thorlabs, New Jersey, USA) and an objective (20×, 0.4 NA, RMS20X, Thorlabs, New Jersey, USA). The light illuminated area on the DMD included 32 × 32 independent input modes, with each 2 × 2 micromirrors grouped as one mode. An objective (20×, 0.4 NA, RMS20X, Thorlabs, New Jersey, USA) and a tube lens (AC254-0100-A-ML, Thorlabs, New Jersey, USA) were used to magnify the output light beam before it was captured by a complementary metal-oxide-semiconductor (CMOS) camera (C11440-22CU01, Hamamatsu Photonics, Shizuoka, Japan) with a frame rate of 200 frames per second (fps) for MMF characterisation.

## 3 Results

### 3.1 Maximising the PBR with the RVITM-based algorithm

**Fig. 3:**
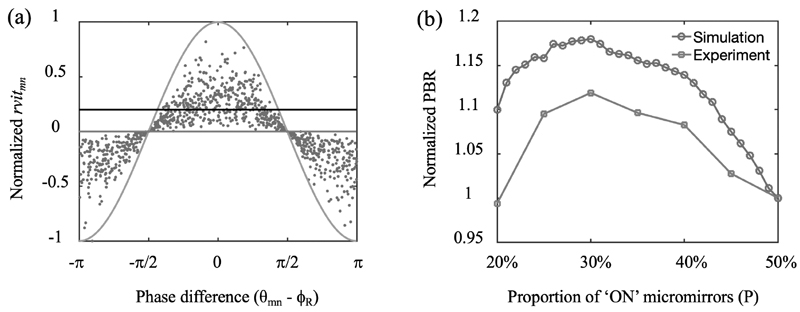
Maximising peak-to-background ratio (PBR) with the RVITM-based algorithm. (a) The relationship between rvit_*mn*_ values and phase difference (*θ_mn_* − *ϕ_R_*) that has an envelope of a cosine function (green curve). When *P* = 50%, the micromirrors with *rvit_mn_* above the red line (*rvit_mn_* = 0) are switched ‘ON’, and when P = 30%, micromirrors with *rvit_mn_* values distributed in the dome region above the black line are switched ‘ON’. (b) PBR as a function of the proportions of switched ‘ON’ micromirrors (P) to the total number of micromirrors with both simulations and experiments.

With the RVITM-based algorithm, each micromirror corresponds a phase difference *θ_mn_* − *ϕ_R_* and a transmission constant *rvit_mn_*. Fig. 3(a) shows the relationship between the *rvit_mn_* value obtained via Eq. 5 and the phase difference obtained from the known TM for each micromirror in numerical simulations. The distribution of *rvit_mn_* values has an envelope of a cosine function (green curve in Fig. 3a). As the phases of *t_mn_* obey an uniform distribution in [−*π*, *π*], approximately half number of the *rvit_mn_* have positive values and their corresponding phase differences *θ_mn_* − *ϕ_R_* are in the range of [−*π*/2, *π*/2]. So, switching ‘ON’ the corresponding micromirrors with positive rvit_*mn*_ values resulted in constructive interference and thus maximised the light intensity at the target output position leading to light focusing. The PBR was strongly dependent on the P value (Fig. 3b). With both experiments and simulations, the PBR increased with P decreasing from 50% (red line in Fig. 3(a)) to 30%, where it reaches the global maximum. Compared to P = 50%, P = 30% led to a lower peak intensity at the focusing position but a higher PBR, suggesting that switching ‘ON’ micromirrors with *rvit_mn_* values distributed in the dome region above the black line in Fig. 3 (a) substantially suppressed the background intensity.

### 3.2 Performance comparison in numerical simulation

**Fig. 4:**
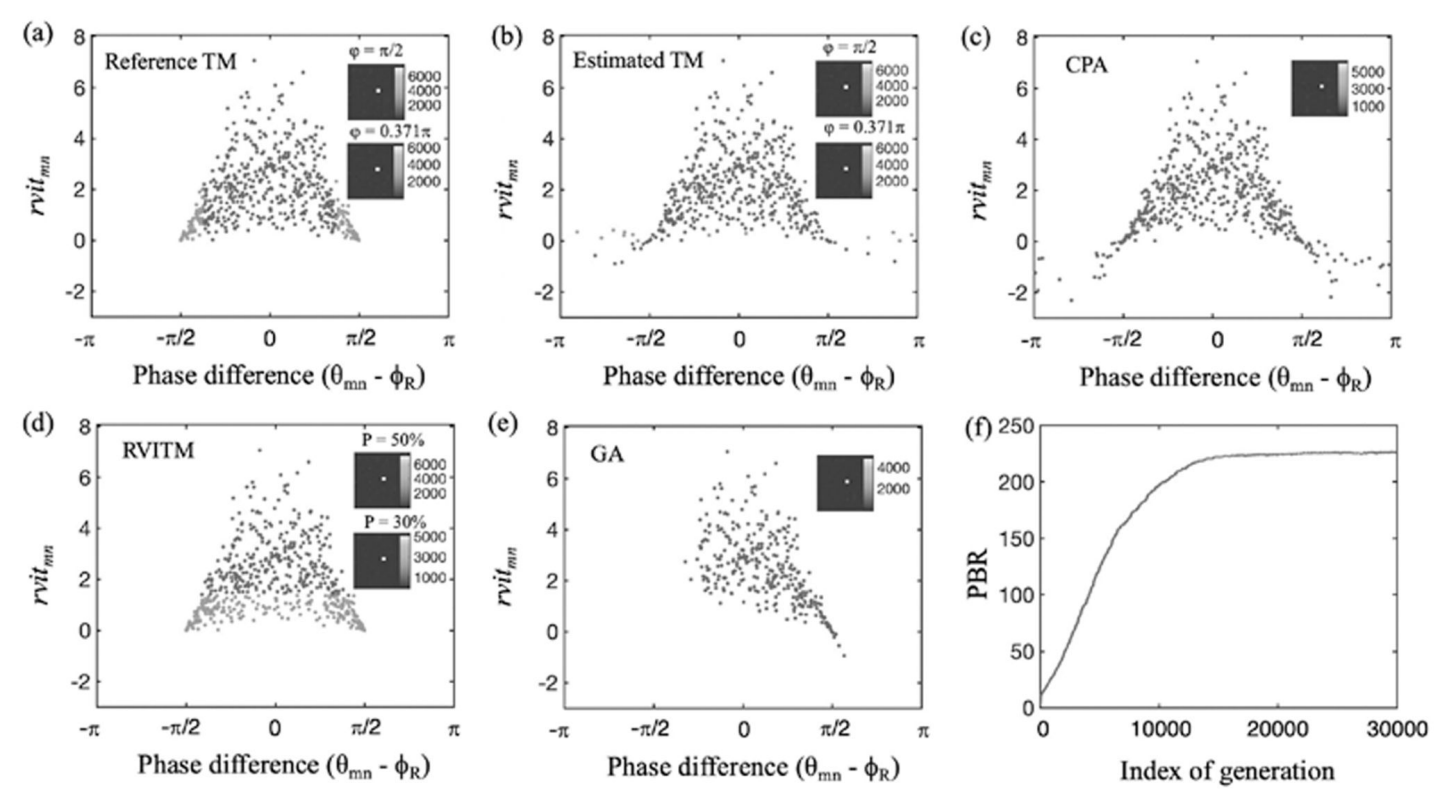
Variations of the DMD micromirrors that are chosen to be switched ‘ON’ for focusing and their corresponding *rvit_mn_* and phase difference (*θ_mn_* − *ϕ_R_*) with different non-holographic algorithms in simulations. (a) Reference TM; when |*θ_mn_* − *ϕ_R_*| ≤ *π*/2, the achieved peak intensity *I_m_* = 7227, and the peak-to-background ratio PBR = 183.0; and when |*θ_mn_* − *ϕ_R_*| < 0.371*π*, *I_m_* = 6182 and PBR = 208.4. (b) TM-based algorithm (estimated TM), when |*θ_mn_* − *ϕ_R_*| < *π*/2, the achieved *I_m_* = 6563 and PBR = 170.4, and when |*θ_mn_* − *ϕ_R_*| < 0.371*π*, the achieved *I_m_* = 6257 and PBR = 190.3. (c) Conditional probability algorithm, the achieved *I_m_* = 5720 and PBR = 156.7. (d) RVITM algorithms, when P = 50%, the achieved I_m_ = 7227 and PBR = 183.0, and when P = 30%, the achieved I_m_ = 4170, PBR = 228.2. (e) Genetic algorithm, the achieved Im = 4835 and PBR = 239.7. Insets are the output light focus patterns at the central position. (f) The evolution curve of the average peak-to-background ratio (PBR) in each generation with the GA. In (a) and (b), both the blue and the green dots represent micromirrors that were switched ‘ON’ with *φ* = *π*/2 used as the criterion, while the blue dots represent additional micromirrors that were switched ‘ON’ when the upper boundary *φ* was changed to 0.371*π*; in (d), both the blue and green dots represent micromirrors that were switched ‘ON’ with P = 50% while the blue dots represent micromirrors that were switched ‘ON’ with P = 30%.

The performance of different algorithms was evaluated in numerical simulations and compared in Table 1. To facilitate the comparison of the resulting optimal DMD patterns for focusing obtained from different algorithms, *rvit_mn_* and *θ_mn_* − *ϕ_R_* values were calculated for all the micromirrors using the ground truth TM. The *rvit_mn_* and *θ_mn_* − *ϕ_R_* values corresponding to all the switched ‘ON’ micromirrors that were determined by different algorithms are plotted in Fig. 4. The known ground truth TM was also used to generated an optimum DMD pattern for focusing as a reference. As shown in Fig. 4(a) and (b), the blue and green dots represent micromirrors that were switched ‘ON’ with *ϕ* = *π*/2 used as the criterion, while the blue dots represent the micromirrors that were switched ‘ON’ when the upper boundary *ϕ* was changed to 0.371*π*. With the reference TM, a PBR value of 208.4 was achieved at *ϕ* = 0.371*π*, which is 13.9% higher than a PBR value of 183.0 obtained with *φ* = *π*/2. This increase of PBR is consistent with the theoretical expectation (13.8%) [32]. The PBR values achieved with the estimated TM using the phase retrieval algorithm were slightly smaller than that achieved with the reference TM (170.4 for *φ* = *π*/2 and 190.3 for *φ* = 0.371*π*, respectively). This slight reduction of PBR values can be attributed to the errors of the TM calculation, which is indicated by the different groups of ‘ON’ micromirrors (Fig. 4b). The CPA produced a smaller PBR than the estimated TM method (PBR = 156.7; Fig. 4c). With the RVITM algorithm (Fig. 4d), a threshold of P = 50% resulted in the same DMD pattern (as represented by the blue and green dots) for light focusing as that obtained from the reference TM and hence the same PBR. When P = 30% was used as the criterion, those micromirrors represented by the blue dots were switched ‘ON’ and the PBR value increased to 228.2, which is even higher than that achieved with the reference TM at *φ* = 0.371*π* (PBR = 208.4). The PBR value achieved with the GA reached the highest value of 239.7 among all the considered algorithms after the evolution of 30,000 iterations (Fig. 4e,f).

To compare the focusing speed of different methods, the average time costs taken for different methods to compute an optimal DMD pattern for focusing over 100 output locations were obtained on a personal computer with a 2.3 GHz Dual-Core Intel Core i5 CPU (see Table 1). Although providing the highest PBR, the GA had the longest computation time of 400 s. In comparison, with 200 iterations the computation time for the TM-based method and the CPA was 15 s and 4 s, respectively. The RVITM-based algorithm calculated the *rvit_mn_* values for all the output positions at the same time, while for each output position the computation time for focusing was 7.5 ms.

### 3.3 Performance comparison in experiments

**Fig. 5:**
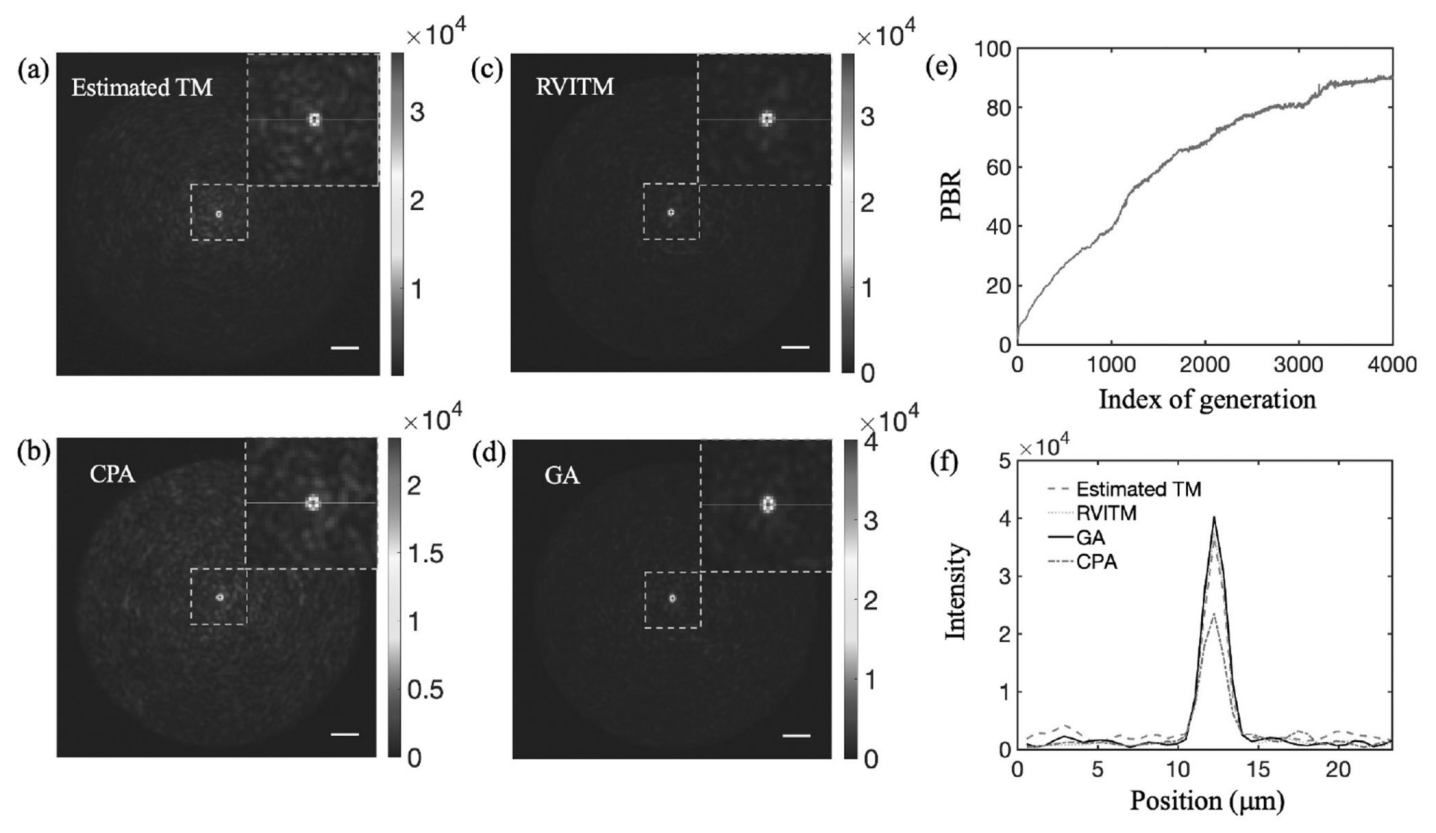
Experimentally obtained light focusing patterns through a multimode fibre with (a) TM-based algorithm (estimated TM) at |*arg*(*t_mn_*)| < 0.371*π*, (b) Conditional probability algorithm (CPA), (c) RVITM-based algorithm at P = 30% and (d) Genetic algorithm (GA). (e) The evolution curve of the peak-to-background ratio (PBR) with the GA over 4000 iterations. (f) The intensity profiles of the achieved light foci along the red lines for different methods. Scale bar, 10 *μ*m.

The output light intensity patterns of an optical focus generated at the distal end of the MMF using different methods are shown in Fig. 5. With the estimated TM, the PBR was 58.5 when switching ‘ON’ micromirrors with |*arg*(*t_mn_*)| < *π*/2, and increased to 64.2 with |*arg*(*t_mn_*)| < 0.371*π* (Table 1). While the PBR achieved with the CPA was the lowest (36), the GA produced the largest PBR of 91 among all the algorithms after 4000 iterations (Fig. 5d,e). The relationship between the achieved PBR and P with the RVITM-based algorithm is shown in Fig. 3 (b). The same as in simulations, the highest PBR value of 89.4 was reached at *P* = 30% (Fig. 5c) compared to a PBR of 79.9 at *P* = 50%. The profiles of light foci are compared in Fig. 5(f), the foci achieved with different methods had approximately the same diameter of ~1.7 *μ*m. The estimated TM had a higher background intensity compared to the reference TM, GA and RVITM methods, which can be attributed to the difference between the estimated TM and the reference TM. In the experiments, the signal-to-noise ratio (SNR) was estimated as 33.3. We also studied the influence of noise in simulations by setting SNR from 33.3 to 3.33, which are lower than the SNR obtained in experiments. The PBRs achieved with all the algorithms were very close to values achieved without noise, indicating that the SNR in this range has neglectable impact on the performance of the algorithms. The deviation of PBRs between simulation and experimental results may be attributed to several factors including the system instability, the fluctuation of laser mode and energy, the non-uniformity of the laser beam and the light coupling loss due to the diffraction of the DMD. The GA had the longest focusing runtime of ~2.2 h, while the time cost was 46 s, 36 s and 10.3 s for the estimated TM, CPA and the RVITM algorithms including both time costs for input DMD pattern displays and data processing, respectively.

## 4 Discussion

In this study, we investigated the performance of four representative non-holographic DMD-based methods for focusing light through a MMF, including the RVITM algorithm, an estimated TM-based algorithm, the CPA and a GA. Although the RVITM method has been used for wavefront shaping based on photoacoustic signals as feedback [25], in this study, it is the first time that it is used for MMF-based endoscopy applications. With both numerical simulations and experiments for focusing light through a MMF, we studied the maximum achievable PBR for different methods and compared them with theoretical expectations. We demonstrated that the PBR of the optical foci can be further improved by switching ‘ON’ micromirrors corresponding to |*arg*(*t_mn_*)| < 0.371*π* compared to that achieved with the commonly used criterion *real*(*t_mn_*) > 0 for DMD-based WS [22, 23, 32].

Different from methods relying solely on phase information, the RVITM combines both phase and amplitude information and achieved a higher PBR at *P* = 30% as compared to the estimated TM method, indicating that the enhancement of the PBR can benefit from employing amplitude information. Additionally, we found that the GA achieved the highest PBR among all the investigated methods via a large number of iterations as expected. Interestingly, a small number of micromirrors with negative *rvit_mn_* values were switched ‘ON’ with the GA (Fig. 4e), indicating that although these micromirrors had negative contributions to the light intensity at the focusing position, they may have substantially reduced the average intensity of the background, leading to a higher PBR compared to those methods considering only micromirrors with positive *rvit_mn_* values. However, the GA cost a much longer time for fibre calibration and hence hindered its use in practical applications where repeated characterisations are required. It should be noted that the parameter settings of the GA can affect the performance and hence the PBR and computation time for the GA might be further improved. The improved RVITM algorithm in this study provided a practical solution to further increase the PBR by setting a threshold based on the *rvit_mn_* values. In future works, the relationship between the PBR and the phase and amplitude of the complex-valued TM could be investigated. To further increase the PBR, more independent micromirrors could be used in the future, however, at the expense of the characterisation time [23].

Changes in the fibre geometry and variations in the environmental temperature can lead to substantial changes in light transmission characteristics of MMFs and hence degraded focusing performance, which is a primary obstacle for the translation of MMF-based endoscopes for biomedical applications. Several approaches were proposed to address this challenge. In a study by Ploschner *et al*. [1], a complex-valued TM was reconstructed after the fibre bending with the knowledge of the MMF geometry. However, this approach is limited to short fibres with simple bending [1]. The same group also reported that gradient-index MMFs have a higher resistance to geometry changes compared to step-index MMFs, and demonstrated that the imaging performance with gradient-index MMFs was only slightly degraded after fibre bending [36]. Repeated fibre characterisation could also be a potential solution to address the challenges involved with fibre bending for biomedical applications, and as such high-speed fibre characterisation methods such as the RVITM algorithm could be advantageous. In addition, the employment of high-performance computing can also shorten the characterisation time [37].

This study was focused on non-holographic approaches that benefit from a lower system complexity, higher temporal stability compared to holographic approaches. It is worthy noting that some of reported holographic approaches could be advantageous for specific applications. For example, DOPC was studied for focusing through a dynamic tissue within several milliseconds [38] owing to ultrafast data acquisition and processing. Furthermore, the uniform light enhancement across all the spatial locations on the output fibre facet was achieved with DOPC methods [7] and the TM method using an external reference light arm for TM measurement [2]. An off-axis Lee hologram approach [2, 11] was also studied with a DMD for phase modulation, achieving a high enhancement factor of 3800 for focusing light through a MMF with a diameter of 50 μm and a numerical aperture of 0.22. However, the diffraction efficiency of the light energy from the employed order was reported to be only 8% [39]. In contrast, non-holographic approaches are more energy efficient as they make use of a larger proportion of the light reflected from the DMD, which could be useful for imaging applications where the availability of suitable light sources is restricted by a trade-off between the light energy and repetition rate, such as photoacoustic imaging [18, 40]. As a burgeoning method, neural networks have also been demonstrated for focusing light at a single focus and projecting heterogeneous patterns with a LC-SLM [41, 42]. Most recently, a hybrid method that combines neural networks and a GA was reported, in which a neural network was used to produce a DMD pattern, which was subsequently used as the first generation in a GA to further improve the light enhancement factor so that the computation time was reduced compared to pure GA-based methods [43].

## 5 Conclusions

In conclusion, we compared the performance of several representative non-holographic algorithms for focusing light through a MMF. The maximum achievable PBR was explored with both numerical simulations and experiments on a simple setup in comparison to theoretical expectations, and was further improved for the RVITM-based algorithm compared to that was achieved by simply switching ‘ON’ micromirrors with positive *rvit_mn_* values in previous studies. It was further found that the GA offered the highest PBR but suffered from a slow computation speed, while the RVITM algorithm provided a comparable PBR to that of the GA but had the highest focusing speed among all the methods investigated.

## Figures and Tables

**Table 1:** Performance of non-holographic algorithms for focusing light through a multimode fibre.

Algorithm	PBR (S)	Runtime (S)	PBR (E)	Runtime (E)^d^	No. of input modes
Reference TM^a^	183.0 208.4	-	-	-	1024
RVITM^c^	183.0 228.2	0.0075 s	79.9 89.4	10.3 s	1024
Estimated TM^b^	170.4 190.3	15 s	58.5 64.2	46 s	1024
CPA	156.7	4s	36	36 s	1024
GA	239.7	400 s	91	7920 s	1024

a,b With reference TM and estimated TM, the PBR refers to two different conditions for determining the optimal DMD patterns for focusing: (|*arg*(*t_mn_*)| < *π*/2 (top) and |*arg*(*t_mn_*)| < 0.371*π*) (bottom).

c For RVITM, the PBR refers to *P* = 50% (up) and *P* = 30% (down). S, simulations; E, experiments.

d In experiments, the time cost includes the time for DMD patterns display during the fibre characterisation and the computation time for producing the optimal DMD patterns, while in simulations, only the latter was included in the time cost.
